# A Wireless Acoustic Emission Sensor System with ACMD-IGWO-XGBoost Algorithm for Living Tree Moisture Content Diagnosis

**DOI:** 10.3390/plants12030601

**Published:** 2023-01-29

**Authors:** Zenan Yang, Yin Wu, Yanyi Liu

**Affiliations:** Department of Internet of Things Engineering, College of Information Science and Technology, Nanjing Forestry University, Nanjing 210037, China

**Keywords:** moisture content, wireless acoustic emission sensor, multi-strategy joint optimization, improved grey wolf optimizer, XGBoost algorithm, living tree trunk

## Abstract

Trunk water has an important influence on the metabolism and ecological balance of living trees, which affects the vegetation growth and moisture cycle of the whole forest ecosystem. The accurate and real-time measurement of moisture content (MC) is of vital guiding meaning to living tree cultivation and forest management. In this paper, a water content diagnosis system based on a wireless acoustic emission sensor network (WASN) was designed and implemented with the aim of the nondestructive detection of water content in living wood trunks. Firstly, the acoustic emission (AE) signal of the trunk epidermis was sampled at high speed; then, its characteristic parameters were calculated and transmitted wirelessly to the gateway. Furthermore, the optimal characteristic wavelet sequence was decomposed by the adaptive chirp mode decomposition (ACMD), and the improved grey wolf optimizer (IGWO) optimization XGBoost established the MC prediction model, which was improved by the multi-strategy joint optimization. Finally, field monitoring was carried out on Robinia Pseudoacacia, Photinia serrulata, Pinus massoniana and Toona sinensis. The average diagnostic accuracy reached 96.75%, which shows that the diagnosis system has excellent applicability in different working conditions.

## 1. Introduction

Forests are the largest ecosystem on earth and have the functions of regulating the climate, preventing wind and fixing sand, and mitigating the greenhouse effect [1]. However, in the last couple of years, extreme drought and high-temperature events have increased significantly, leading to frequent forest decay and tree death on a global scale. The research on the mechanism of plant drought death has become a hot topic, among which the analysis of the water physiology and water content of woody plants is the core content [2]. The current remote sensing measurement methods cannot accurately detect the physiological state of a single tree and cannot meet the needs of the water relationship research, and the traditional method of manual field sampling is too time-consuming and laborious [3]. In this paper, after processing AE signals collected by WASN, a diagnostic model of tree core MC was established based on ACMD-IGWO-XGBoost.

At present, the drying method [4], dielectric constant method [5] and resistance method [6] are the main methods for measuring the moisture content of living wood, but they all have some shortcomings. The drying method needs to collect samples, which will cause damage to living wood, and the process is complicated. When using the dielectric constant method, it is difficult to match the size of the sensor and the trunk, and the power consumption of the detection circuit is high. The resistance method also causes damage to the trunk. However, we pay more attention to the application of real-time, convenient and lossless AE signals in measuring MC [7]. Yang Li et al. have found that the AE characteristics of Yunnan pine with different MC are very different [8]. Xiaosong Li used wavelet decomposition to analyze AE signals in the frequency domain [9]. Llana et al. tested the nondestructive testing methods (AE, vibration sensors and probes) of conifer species such as Pinus kiwi and Pinus scotchinensis with different water content and deduced the corresponding regression correction coefficient [10]. 

In addition, traditional signal decomposition methods (EMD, VMD, and ICEEMD) have been applied to other industries, such as power load prediction [11], carbon dioxide emission prediction [12], etc. ACMD is mostly used for fault identification of mechanical bearing structures because of its superiority in handling compared with primitive, unsteady AE [13,14,15]. Govind Vashishtha et al. demonstrated a new method for identifying defects in centrifugal pump impellers through ACMD [16]. Chuancang Ding and Baoxiao Wang proposed an improved Sparsity-ACMD for the detection of rub-impact faults [17]. Xiaolong Wang et al. introduced an ACMD processing technology into wind turbine bearing fault diagnosis for the first time [18]. The existing research results all show the excellent superiority and accuracy of ACMD in the feature recognition of AE signals. However, no research reports have been found on the inversion of the water content of living wood through child wave signs based on ACMD signal decomposition.

**Figure 1:**
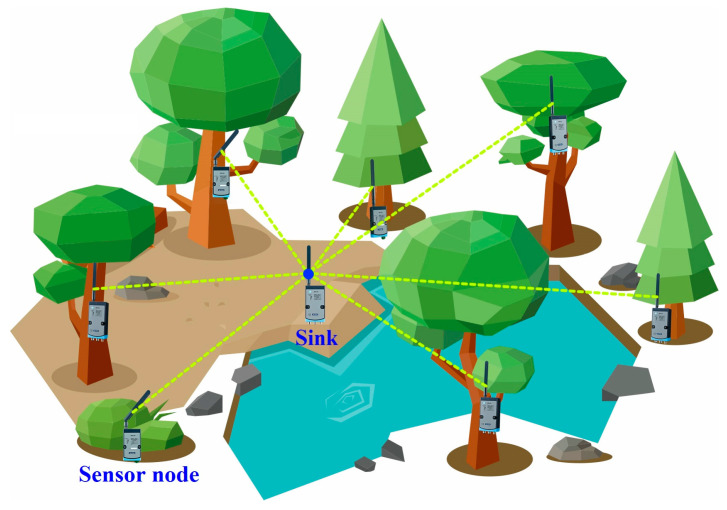
Schematic diagram of WASN.

In this paper, low-power and high-precision wireless acoustic emission nodes designed by ourselves are used to collect the fundamental water content of standing trees. Based on the ACMD of the original AE signal, for the first time, the optimal MC diagnosis model of living standing trees is established by using machine learning-related technologies so as to realize the remote real-time nondestructive monitoring of the MC of standing trees, providing a new mentality for measuring the MC of standing trees. The schematic framework of the WASN is properly displayed in Figure 1.

## 2. System and Methods

### 2.1. Wireless Acoustic Emission Sensor Network

The WASN node used in this paper was further improved on the previous basis. It had two independent AE data acquisition channels and was equipped with a high-speed amplifier, the OPA627, due to the low amplitude of AE signals. Due to the need for low-power wireless transmission, the ultra-low-power digital-to-analog converter AD7536 was responsible for converting and transmitting the collected data to the STM32F405RG central controller, and the LoRa module SX1278 was used to construct the 433MHz wireless communication network.

**Figure 2:**
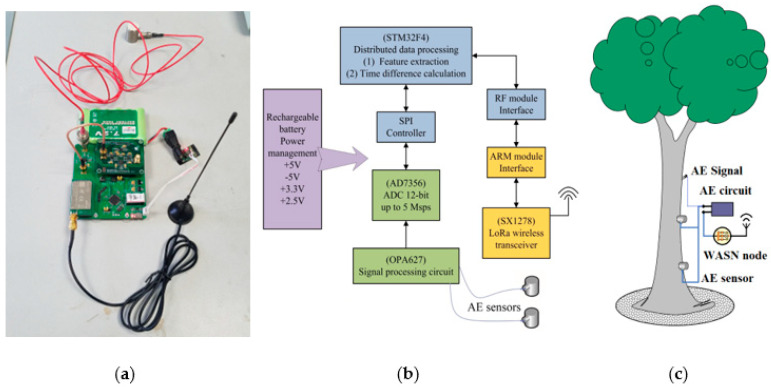
Field testing structure of WASN and its sample node: (**a**) sample of AE node; (**b**) architecture of the wireless AE node; (**c**) site installation diagram.

The excitation sound source for data collection was generated by the regular periodic vibration of the Arduino miniature vibration motor. The AE wave during the process was propagated directly through the surface of the tree trunk and collected by the AE sensor coupled with Vaseline on the surface of the bark. To further strengthen the anti-interference function of this system, two R15α probes were positioned longitudinally at a distance of 10 cm and 20 cm between the acoustic source and the tree at the diameter of breast height (1.3 m above the ground). In this way, the difference term can be calculated for the far and near probes to reduce the measurement error of the system. Four types of trees growing in the same area were selected as sample trees, namely Buxus sinica, Zelkova serrata, Pinus sylvestris and green maple, with a total of 12 trees. The architecture of the WASN and field installation diagram are displayed in Figure 2.

### 2.2. Time Series Decomposition of AE Waveform Based on ACMD

The AE waveform of a tree trunk is affected by its material, shape, moisture and other factors, showing certain characteristics of fluctuation and non-stationarity. However, due to the regular excitation of the micromotor, the AE waveform also has an obvious trend of periodic fluctuation. If the algorithm model can capture the periodic rule of AE waveforms well, the accuracy of the prediction of tree trunk MC will be greatly improved. In this paper, the original non-stationary AE sequence is decomposed by the ACMD algorithm. ACMD is an adaptive signal decomposition algorithm, and the framework matching and tracking of a recursive algorithm can effectively process multimodal signals with time-varying characteristics.

A non-stationary AE signal with *s* components can be defined as:

(1) $$y(t)=\sum_{i=1}^{s}y_{i}(t)=\sum_{i=1}^{s}A_{i}(t)\cos(2\pi \int_{0}^{t}f_{i}(\tau )d\tau +\varphi_{i})$$

where $A_{i}(t)$, $f_{i}(\tau )$ are the instantaneous amplitude and frequency of the *i*-th component. $\varphi_{i}$ represents the initial phase. ACMD is based on the broadband signal through modulation and modulation to narrowband signal to achieve the simplification of solving the problem. Through demodulation technology, the above equation can be converted to:

(2) $$y(t)=\sum_{i=1}^{s}m_{i}(t)\cos(2\pi \int_{0}^{t}{\tilde{f}}_{i}(\tau )d\tau )+n_{i}(t)\sin(2\pi \int_{0}^{t}{\tilde{f}}_{i}(\tau )d\tau )$$

(3) $$m_{i}(t)=A_{i}(t)\cos(2\pi \int_{0}^{t}\left(f_{i}(\tau \right)-{\tilde{f}}_{i}(\tau ))d\tau +\varphi_{i})$$

(4) $$n_{i}(t)=-A_{i}(t)\sin(2\pi \int_{0}^{t}\left(f_{i}(\tau \right)-{\tilde{f}}_{i}(\tau ))d\tau +\varphi_{i})$$

where $m_{i}(t)$ and $n_{i}(t)$ represent the demodulation operator, and $\tilde{f_{i}}(t)$ is the corresponding frequency function. We can see from the above equation that if we choose the suitable $\tilde{f_{i}}(t)$ to make $\tilde{f_{i}}(t)=f_{i}(t)$, the narrowest bandwidth pure amplitude modulated signal is obtained in this ideal case. Each component of the signal is estimated by solving the demodulation bandwidth minimization problem. At this time, ACMD extracts each component of the signal one by one based on the recursive framework, it is converted into the optimization problem of finding the *i*-th signal component.

(5) $$\underset{m_{i}(t),n_{i}(t),{\tilde{f}}_{i}(t)}{\min}\left\{{\left\|m_{i}{}^{\prime \prime }(t)\right\|}_{2}^{2}+{\left\|n_{i}{}^{\prime \prime }(t)\right\|}_{2}^{2}+\lambda {\left\|y(t)-y_{i}(t)\right\|}_{2}^{2}\right\}$$

where ${\left\|•\right\|}_{2}$ is Euclidean norm, $m_{i}{}^{\prime \prime }(t)$ and $n_{i}{}^{\prime \prime }(t)$ are the second derivatives of the demodulation operator, and λ represents the penalty factor. Considering the discrete form of the signal, under the time series of $t=t_{0},\cdots ,t_{N-1}$, the discrete form of the above equation is:

(6) $$\underset{B_{i},D_{i}}{\min}\left\{{\left\|\Lambda B_{i}\right\|}_{2}^{2}+{\left\|n_{i}{}^{\prime \prime }(t)\right\|}_{2}^{2}+\lambda {\left\|y(t)-G_{i}B_{i}\right\|}_{2}^{2}\right\}$$

where Λ = diag[Ω, Ω] is the square diagonal matrix, Ω is a second-order difference matrix, and *G_i_* is the frequency diagonal matrix in the discretized form *B_i_* = [*m_i_*^T^, *n_i_*^T^]^T^.

In the decomposition of the original AE signal step by step using the demodulation bandwidth minimization problem, the first signal component is subtracted from the original signal component and looped several times until all signal components are obtained.

### 2.3. Grey Wolf Optimizer (GWO)

The algorithm principle of GWO is to imitate the social class system and prey hunting track of the grey wolf, and it achieves the purpose of optimization according to the searching, surrounding and hunting behavior in the process [19]. The grade divisions, which occur from high to low and are usually divided into four levels, are α, β, δ, and ω.

The leader of the pack is defined as the α wolf, subordinates who assist the α wolf in making decisions are defined as β wolves, the third rank of wolves are known as δ wolves, and the lowest rank are the ω wolfs, which are subject to all other wolves of all classes. It may not seem as though ω-wolves have an important role, but they are essential to the internal balance between the species.

The mathematical model of the behavior of a grey wolf group to search for the location of prey and surround it can be expressed as:

(7) $$D=E\times X_{p}(t)-X(t)$$

(8) $$X_{i}(t+1)=X_{p}(t)-A\times D$$

where D represents the distance vector between the prey and grey wolves,t represents the current number of iterations,$X_{p}(t)$ is the vector of the current grey wolf population’s position, and E and F are both the coefficient vectors, the calculation of which is as follows:

(9) $$F=2u\times r_{1}-u$$

(10) $$C=2r_{1}$$

(11) $$u=2(1-\frac{t}{t_{\max}})$$

where $t_{\max}$ is the maximum iterations number; $r_{1}$, $r_{2}$ are vectors randomly ranged in [0, 1]; and a is the distance control parameter with an initial value $a_{0}$ = 2.

In the process of grey wolves hunting prey, the α wolves lead the pack to search for the prey. Then, led by the alpha, the β and δ wolves attack and catch their prey. In the process, α, β, δ wolves are nearest to the prey position, and the position of the three wolves can be used to calculate the position of the individual grey wolf moving toward the prey:

(12) $$D_{\alpha }=\left|E_{1}\times X_{\alpha }(t)-X(t)\right|$$

(13) $$D_{\beta }=\left|E_{2}\times X_{\beta }(t)-X(t)\right|$$

(14) $$D_{\delta }=\left|E_{3}\times X_{\delta }(t)-X(t)\right|$$

The advance direction and step size of the ω wolves relative to the α wolves, β wolves and δ wolves are calculated as:

(15) $$X_{1,\omega }(t+1)=X_{\alpha }(t)-F_{1}\times D_{\alpha }$$

(16) $$X_{2,\omega }(t+1)=X_{\beta }(t)-F_{1}\times D_{\beta }$$

(17) $$X_{3,\omega }(t+1)=X_{\delta }(t)-F_{3}\times D_{\delta }$$

The final position vector of the ω wolves is:

(18) $$X_{\omega }(t)=\frac{X_{1,\omega }+X_{2,\omega }+X_{3,\omega }}{3}$$

According to the above model, wolves evolved to surround their prey by constantly updating their positions until they reached a maximum number of iterations or solution accuracy.

### 2.4. Multi-Strategy Joint Optimization of Improved GWO

When solving the problems of function optimization, the GWO algorithm generally uses initial overall information that is randomly generated and characterized by difficulty in retaining the diversity of the population. The quality of the original population greatly affects the convergence speed and global optimization performance of the algorithm.

In order to enhance the global search capability of GWO, avoid results that occur from falling into local optima, and accelerate the convergence rate, the following strategies are adopted for joint optimization: Hénon mapping was used to initialize the gray wolf population so that individuals were evenly distributed in the search space as much as possible, and the diversity of the initial population was increased; the spiral predation strategy was applied to the location updating process of the ω wolf to give consideration to both local exploitation and global search capabilities; and the firefly disturbance strategy was used to perturb the locations of all gray wolves to strengthen their ability to jump out of the local optimal solution, expand their grabble scope, and improve their global search ability.

#### 2.4.1. Hénon Mapping

A chaotic system has both non-convergence and random boundedness. The randomness, regularity and ergodicity of the corresponding chaotic map are suitable for maintaining the multiformity of an algorithm’s population and improving the global grabble ability.

Common chaotic mapping mainly includes tent mapping, logistic mapping and Hénon mapping. A tent map has good ergodic properties, but this map has a fixed point of a rational number, which will cause algorithm failure once the data falls into the fixed-point cycle. Logistic mapping has the disadvantage of an uneven distribution, mainly in the two ranges [0, 0.1] and [0.9, 1], where the probability of a value is much higher than in other ranges. Hénon mapping has the same uniformity as tent mapping but is more stable, which can cover the search blind area to the greatest extent and enhance the individual diversity of gray wolves [20].

Hénon mapping was used to initialize the GWO algorithm population, and the expression was as follows:

(19) $$\left\{ \begin{array}{l} y_{1}(t)=1-a{\left(y_{1}(t-1)\right)}^{2}+y_{2}(t-1)\\ y_{2}(t)=by_{1}(t) \end{array}\right.$$

where *t* is the number of chaotic iterations. When *a* = 0.6 and *b* = 0.7, y_1_(t) and y_2_(t) show chaotic characteristics. Even a small change in the original value will contribute to a great difference in the following chaotic sequence so that the chaotic sequence can spread over the entire search area without repeating.

(20) $$z(t)=abs(\omega y_{1}(t)),i=1,2,\cdots n.$$

We normalized the Hénon sequence from the range [−2, 2.25] to [0, 1] according to the above formula.

(21) $$x_{i}^{t}=lb_{i}+z(t)(ub_{i}-lb_{i})$$

**Figure 3:**
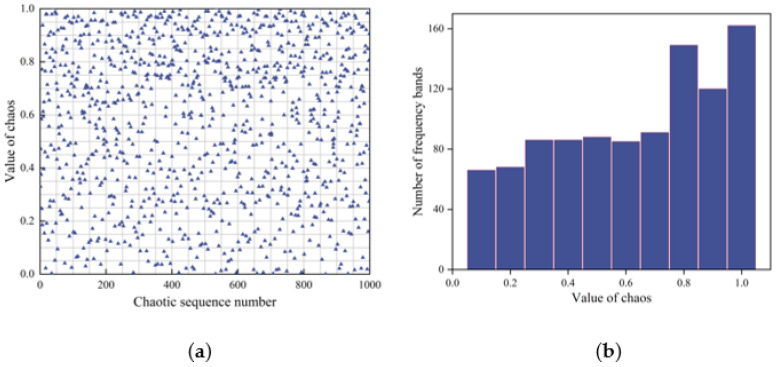
Comparison of chaotic values and random values of Hénon mapping: (**a**) scatter plot of chaotic value dimension distribution and (**b**) chaotic value frequency histogram.

Finally, the initial wolf pack position is obtained through the above equation, where *ub_i_* and *lb_i_* are the maximum bound and minimum bounds of the solution space. It can be seen from the histogram of chaotic value and chaotic value frequency that the chaotic value sequence is distributed evenly in Figure 3.

#### 2.4.2. Reverse Learning Strategy

For the purpose of letting the individual grey wolf find a better solution, the corresponding reverse solution is achieved through the reverse learning strategy, and the fitness values of the current solution and the reverse solution are compared to retain the better solution [21]. The reverse learning strategy is introduced into the grey wolf algorithm, and the mathematical expression is:

(22) $$X_{opbest}(t)=pc+r⊕(qc-X_{best}(t))$$

(23) $$X_{{}_{i,j}}^{t+1}(t)=X_{opbest}(t)+c_{1}⊕(X_{best}(t)-X_{opbest}(t))$$

where $X_{best}(t)$, $X_{opbest}(t)$ are the optimal solution of the *t*-th iteration and the corresponding reverse solution; pc and qc are the maximum and minimum bounds; r is a random number matrix; and $c_{1}$ is the control parameter, the calculation of which is as follows:

(24) $$c_{1}={\left(iter_{\max}-\frac{t}{iter_{\max}}\right)}^{t}$$

#### 2.4.3. Firefly Perturbation Strategy

At the late stage of the GWO iteration, in consideration of the assimilation of individual wolves, the algorithm tends to fall into local optimization. Aiming at this problem, the firefly perturbation strategy was used to perturb the positions of all individual grey wolves [22]. The firefly disturbance strategy is based on firefly brightness to move to a new location. The grey wolf position update formula is thus:

(25) $$Y_{i,J}^{t+1}=X_{i,j}^{t+1}+\sigma (X_{i,j}^{t+1}-X_{p}^{t})+\theta (rand-\frac{1}{2})$$

where σ represents attraction, and $\sigma =\beta_{0}\times e^{-\gamma r_{i,j}^{2}}$; $\beta_{0}$ represents maximum attraction, γ represents the absorption coefficient of the light’s intensity; $r_{i,j}$ is the distance between $X_{i,j}^{t+1}$ and $X_{p}^{t}$; θ is the step factor range in [0, 1]; and rand is a regularly spread random number in [0, 1].

### 2.5. XGBoost

XGBoost is an additional model composed of *k* regression tree models [23]. We set the tree model of the *t*-th iteration training as $f_{t}(x_{j})$. Then, the final prediction result of the *k* regression tree integration became:

(26) $$\hat{y_{i}}=\sum_{k=1}^{K}f_{k}(x_{i}),f_{k}\in F$$

where $f_{k}$ represents an independent regression tree, and F are all the regression trees; $f_{k}(x_{i})$ is the predicted value of sample *i* on the *k*-th regression tree; and $\hat{y_{i}}$ is the final forecast result.

The objective function of XGBoost is defined as:

(27) $$Ob_{j}=\sum_{i=1}^{n}C(y_{i},\hat{y_{i}})+\sum_{k=1}^{K}\Omega (f_{k})$$

(28) $$\Omega (f_{k})=\gamma T+\frac{1}{2}\gamma {\left\|\xi \right\|}^{2}=\gamma T+\frac{1}{2}\eta \sum_{j=1}^{T}\xi_{j}^{2}$$

where $\sum_{i=1}^{n}C(y_{i},\hat{y_{i}})$ is the loss function, $\Omega (f_{k})$ is the regular term, T and ξ represent the number and weight of the leaf nodes, respectively, and γ and η are the penalty terms that inhibit the model complexity caused by the increase in leaf nodes.

Here, $\hat{y_{i}}$ adds a new function $f_{t}$ at the *t* iteration of the greedy optimization target function to improve the precision of the prediction. Here, the target function of the model in the *t* iteration is:

(29) $$J(f_{t})=\sum_{i=1}^{n}(L(y_{i},{\hat{y_{i}}}^{t-1})+f_{t}(x_{i}))+\Omega (f_{t})$$

By expanding the second-order Taylor formula of the loss function in the above equation, the simplified target function of the *t*-th training is:

(30) $$J(f_{t})=\sum_{i=1}^{n}(g_{i}f_{t}(x_{i})+\frac{1}{2}h_{i}f_{t}{}^{2}(x_{i}))+\Omega (f_{t})$$

where $g_{i}$ represents the first-order derivative of the loss function, and $h_{i}$ represents the second-order derivative of the loss function. After substituting (28) into (30), the final target function is obtained:

(31) $$J(f_{t})=\sum_{i=1}^{n}(g_{i}f_{t}(x_{i})+\frac{1}{2}h_{i}f_{t}{}^{2}(x_{i}))+\frac{1}{2}\eta \sum_{j=1}^{T}\xi_{j}^{2}+\gamma T$$

The optimal $\xi_{j}$ is: $\xi_{{}_{j}}^{*}=-\frac{\sum_{i\in I_{j}}g_{i}}{\sum_{i\in I_{j}}h_{i}+\eta }$, and at this point, the optimal target value is obtained:

(32) $$J(f_{t})=-\frac{1}{2}\sum_{j=1}^{T}\frac{{\left(\sum_{i\in I_{j}}g_{i}\right)}^{2}}{\sum_{i\in I_{j}}h_{i}+\eta }+\gamma T$$

where $J(f_{t})$ represents the structure fraction of the tree, and the smaller it is, the higher the precision.

### 2.6. Framework of ACMD-IGWO-XGBoost

**Figure 4:**
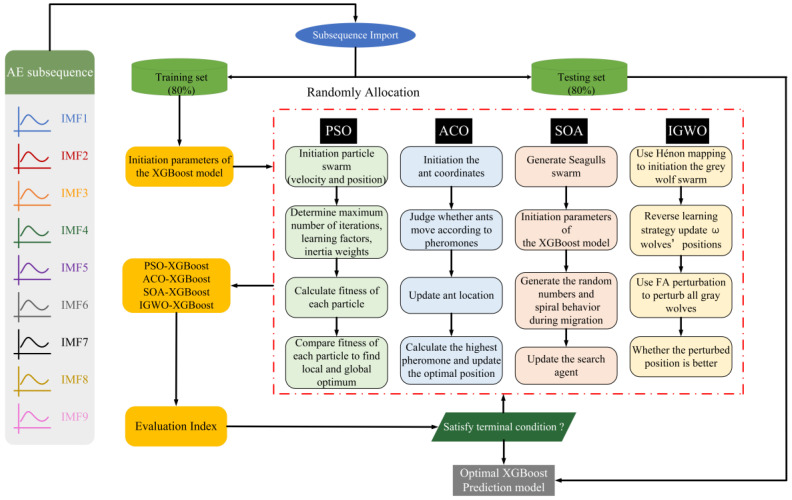
Optimization process of xgboost of four hybrid intelligent models based on ACMD method.

Taking the waveform parameters collected by WASN as the sample set, the figure shows the framework of the water-content diagnosis method for living wood designed in this paper. Firstly, the original AE signal was decomposed by ACMD, and the subsequence waveform was divided into a training set and a testing set based on the score of 8:2. Then, the relevant parameters of XGBoost are initialized, and the heuristic algorithms of particle swarm optimization (PSO) [24], ant colony optimization (ACO) [25], the seagull optimization algorithm (SOA) [26] and IGWO are used to adjust the extreme gradient boost superparameters. Thus, four evaluation indexes were selected to compare the prediction results of IGWO, which was improved by multi-strategy joint optimization in this paper, with the results of the other three algorithms and finally output the optimal diagnostic model of live wood MC. Figure 4 shows the whole analytical process of the different algorithms adopted in this study.

### 2.7. Data Acquisition and Preprocessing

**Figure 5:**
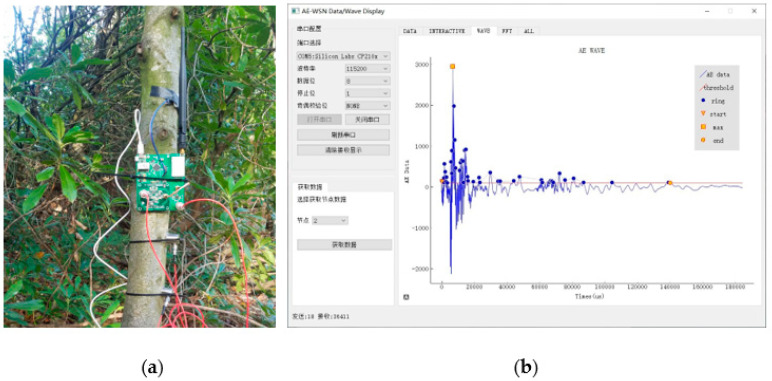
Actual sensor node installation and data collection diagram: (**a**) deployment of AE nodes and (**b**) display interface of AE data.

Figure 5 displays the AE nodes’ on-site diagram and data acquisition interface. As can be seen, AE feature parameters such as amplitude, rise time, duration, ring count and signal energy can all be precisely measured and calculated.

#### 2.7.1. Time Series Decomposition of AE Waveform Signal Based on ACMD

Here, we compare the wave decomposition effectiveness of ACMD and VMD [27]. A total of 90,000 sample points for a consecutive 9 s were selected from the training data, and VMD and ACMD were used for decomposition, as shown in the decomposition diagram below. The ordinate represents the amplitude of the sub-sequence after the original amplitude decomposition of each sampling point.

**Figure 6:**
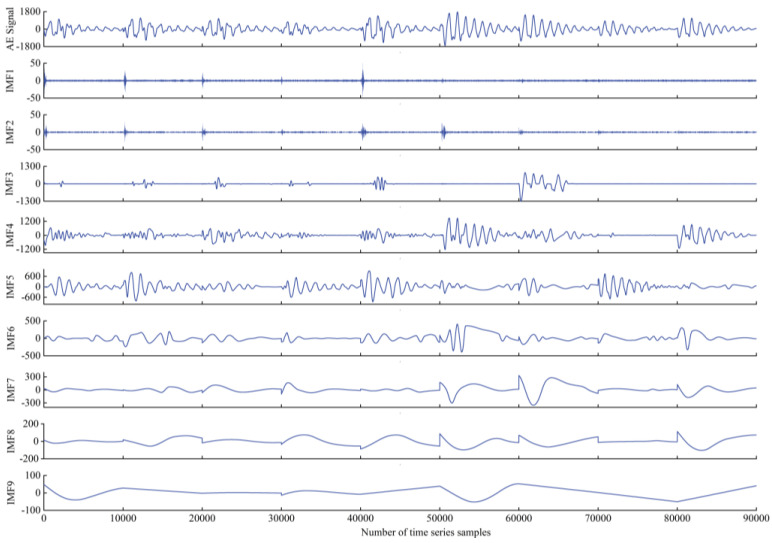
VMD algorithm decomposition results.

**Figure 7:**
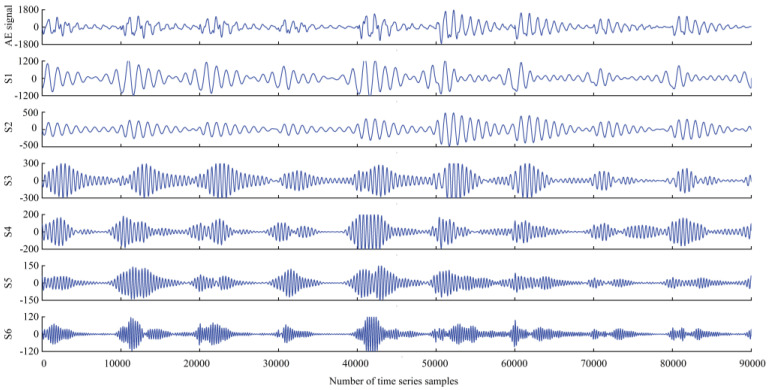
ACMD algorithm decomposition results.

The original AE signal still has a strong time-varying non-stationary property. It can be seen from the waveform decomposition diagram that 9 sequential wavelet waves are generated after VMD decomposition, while only 6 are generated after ACMD decomposition in Figure 6 and Figure 7. While the number decreases, the amount of effective information of a wavelet increases, reducing the amount of subsequent calculations, which highly proves the efficiency and effectiveness of ACMD in AE signal decomposition.

#### 2.7.2. IGWO-XGBoost Performance Test

To further explore the factors affecting IGWO’s performance regarding multi-strategy joint optimization, Hénon mapping, reverse learning strategy and firefly disturbance were, respectively, used for optimization comparison experiments, and set three optimization policies. They were named IGWO_1_, IGWO_2_, and IGWO_3_. Details are in Table 1.

**Figure 8:**
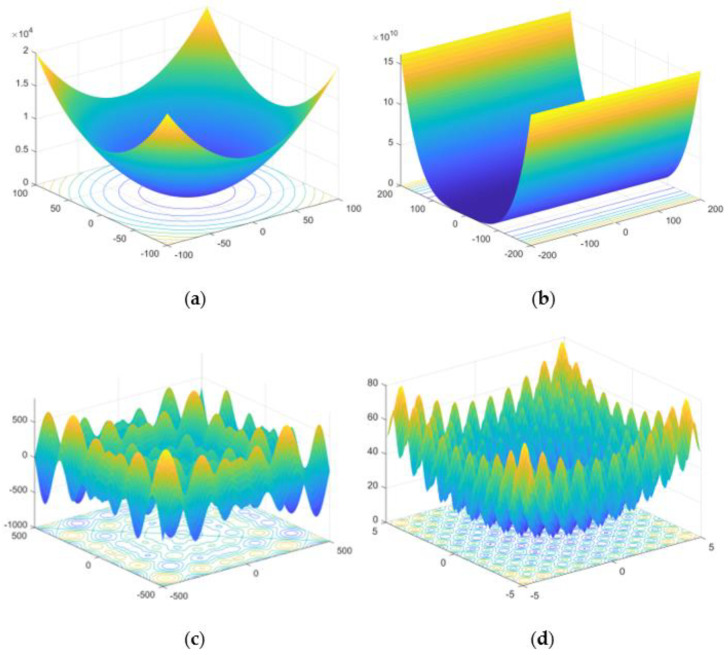
Standard test function: (**a**) sphere function; (**b**) Rosenbrock function; (**c**) Schewfel function; (**d**) Rastrigin function.

Here, as shown in Figure 8, we selected the Rosenbrock single-peak function, sphere single-peak function, Schwefel multipeak function and Rastrigin multipeak function as test functions, respectively.

During the test, in order to remove errors caused by accidental factors, 50 independent experiments were conducted on the 5 optimization algorithms, respectively, and the final results were averaged. The quantity size was set to *n* = 30, and the maximum number of iterations were T = 500. Here in IGWO, ε = 8, b1 = 0.6, and b2 = 0.6.

**Figure 9:**
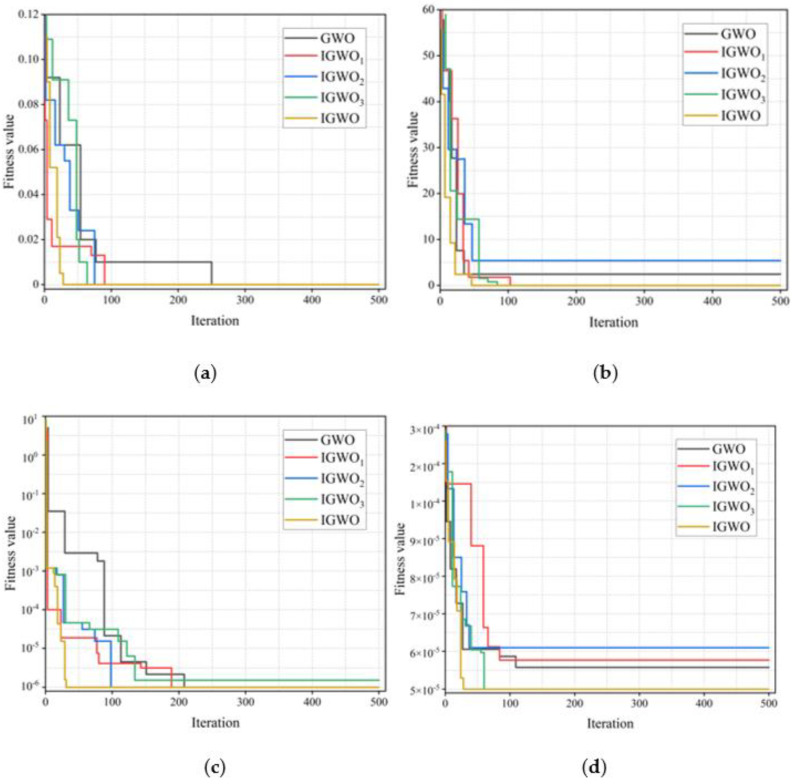
Different GWO algorithms converge on four standard test functions: (**a**) sphere function; (**b**) Rosenbrock function; (**c**) Schewfel function; (**d**) Rastrigin function.

In Figure 9, when the sphere standard function test is adopted, all four optimization algorithms can complete iterative convergence within 200 times and successfully search for the minimum value, which also means that compared with the basic GWO algorithm, the three improvement strategies cooperate to work together on GWO. The other three optimization strategies all fall into local optimality, and the convergence rate decreases under different test functions. IGWO_2_ without the reverse learning strategy fell into local optima twice, indicating that the reduction of species diversity would cause the optimization algorithm to become caught in local optima. On the other hand, it shows that the reverse learning strategy plays a leading role in the improvement of IGWO performance, and Hénon mapping and the firefly perturbation strategy coordinate convergence speed and the global search ability of the algorithm.

IGWO does not fall into local optimality in all test functions, showing excellent global search performance and fast convergence, indicating that IGWO’s global optimization ability and convergence speed are superior to GWO, IGWO_1_, IGWO_2_ and IGWO_3_.

#### 2.7.3. Comparative Analysis of Different Improvement Algorithms

Based on XGBoost’s low requirements and high performance in solving problems and for waveform feature processing, heuristic algorithms such as PSO, ACO, SOA and IGWO are used to optimize the extreme gradient boost hyperparameters. XGBoost is the baseline model for predicting MC. However, the XGBoost algorithm has too many parameters, and the regular terms are constantly adjusted in the optimization process, so it is hard to select the optimal parameter value of the heuristic optimization algorithm in advance. Using random numbers within the valid range of a particular parameter of a heuristic algorithm can better optimize the result. First, we initialized the relevant hyperparameters of the XGBoost model. Then the swarm sizes were set to 50, 100, 150, 200, 250, and 300. Finally, to further verify the prediction performance of these models, multiple indicators of root-mean-square error (RMSE), mean absolute error (MAE), mean absolute percentage error (MAPE), and goodness of fit (R^2^) were employed for the evaluation.

**Figure 10:**
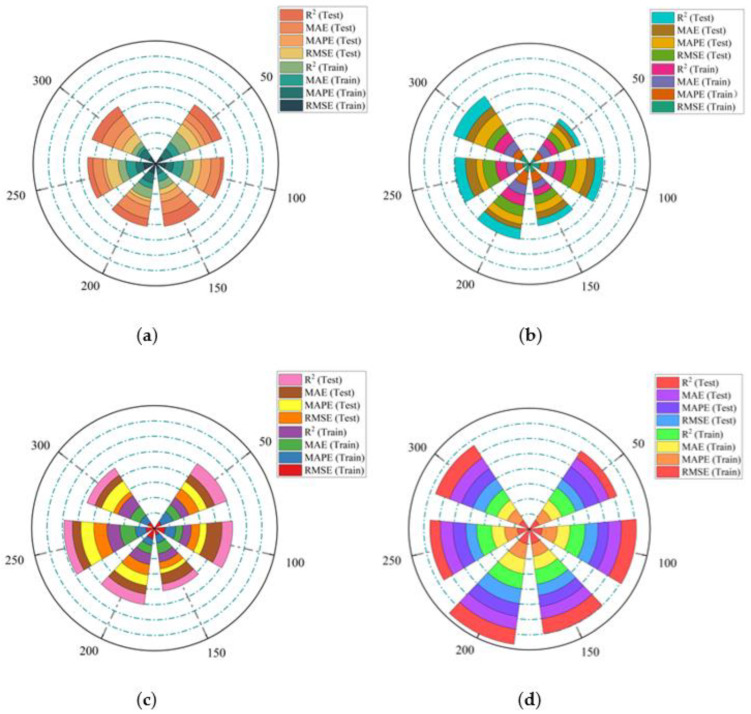
Comparison of evaluation indexes for MC prediction algorithms: (**a**) PSO-XGBoost; (**b**) ACO-XGBoost; (**c**) SOA-XGBoost; (**d**) IGWO-XGBoost.

In Figure 10, we stipulate that the four final algorithms shall be sorted according to the value of the population according to the evaluation index, including the evaluation index of the training set and the evaluation index of the testing set. The wider the color block in the figure, the better the evaluation index. IGWO-XGBoost has the best performance in the 6 types of swarms: 50, 100, 150, 200, 250 and 300. In particular, when the swarm size is 200, the best parameter combination and the optimum performance can be achieved. Considering the complex correlation between every decomposed waveform and MC, this result is very satisfactory. Therefore, in the subsequent field measurement experiments, the total swarm was set to 200.

## 3. Result

**Figure 11:**
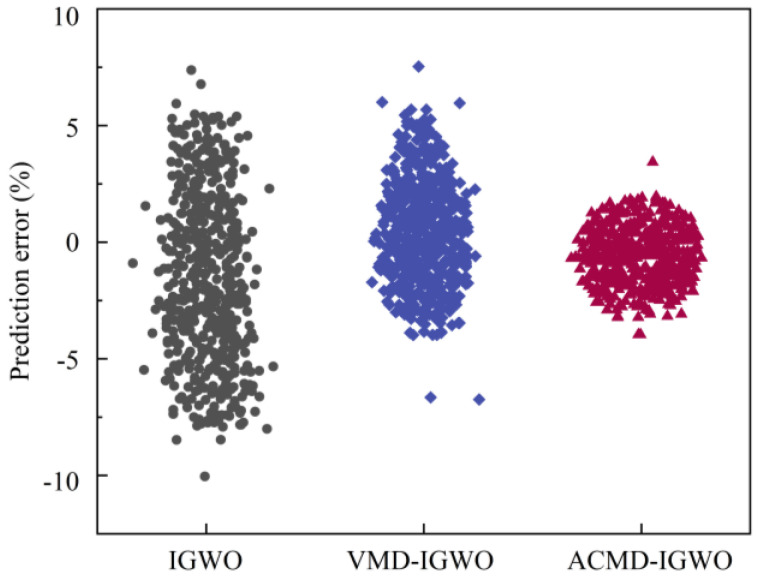
Prediction error distribution diagram using different decomposition methods.

Five hundred groups of original AE waveforms in the same tree were randomly selected, and IGWO after multi-strategy joint optimization was used for predictive modeling of AE signals without decomposition, VMD decomposition and ACMD decomposition. Figure 11 shows the prediction errors of each method.

Compared with the IGWO model, which used the original AE signal for prediction without decomposition, VMD-IGWO and ACMD-IGWO reduced the prediction error while the number of prediction deviation points also decreased, and ACMD-IGWO even reduced by nearly 118.4%. The prediction error distribution is concentrated near the origin, so the prediction error is smaller, and the evaluation index value is better. The results show that the subsequences after ACMD decomposition have a stronger tendency, which is more conducive to improving the prediction accuracy.

**Figure 12:**
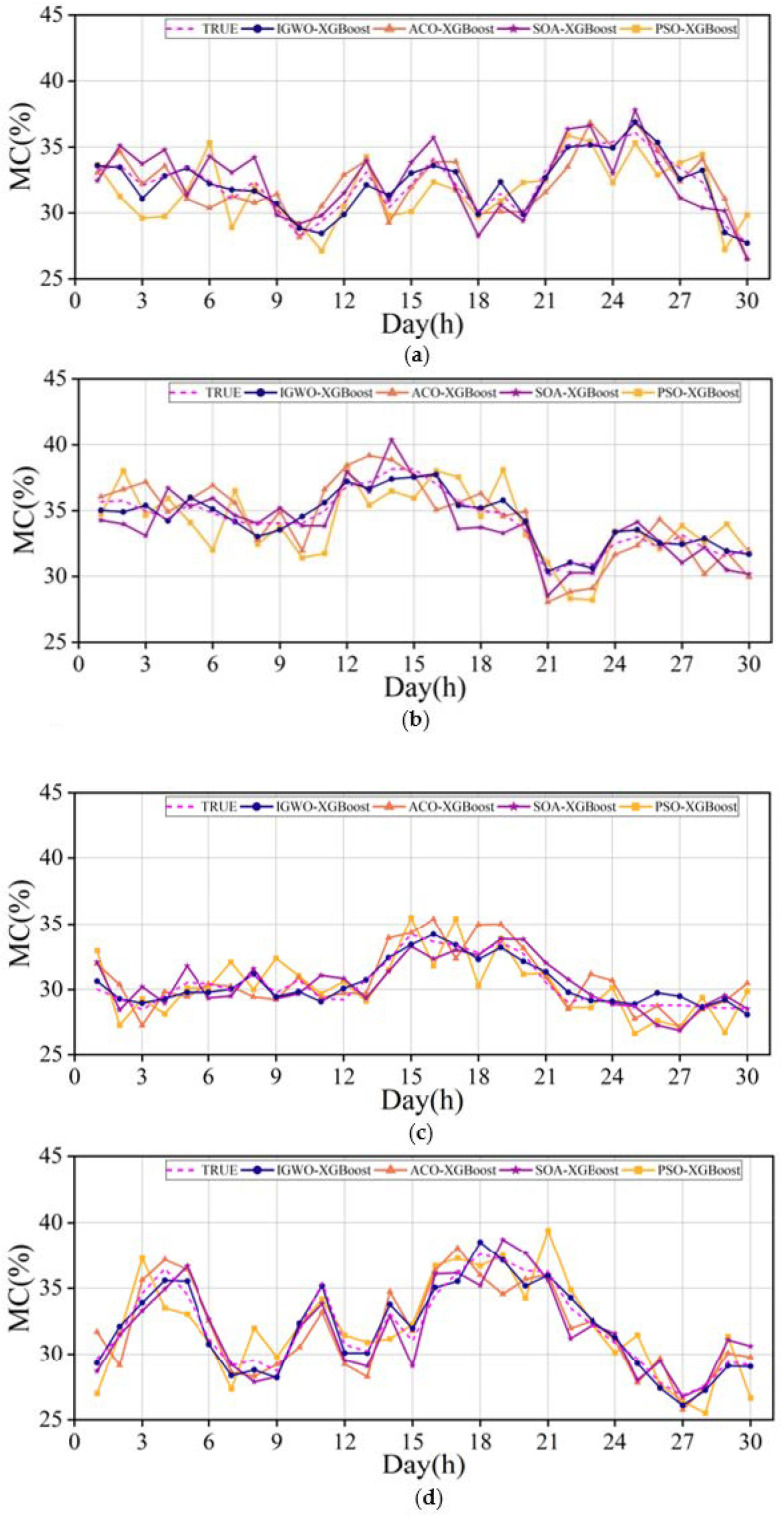
Trunk MC variations of 4 selected trees during 30 days: (**a**) Robinia Pseudoacacia; (**b**) Photinia serrulata; (**c**) Pinus massoniana; (**d**) Toona sinensis.

The MC of a standing tree trunk is an important parameter to measure the physiological condition of this tree. However, the MC of the trunk of living trees changes dynamically in real time. We expect that the IGWO-XGBoost in this paper can be used to predict the MC of different tree species with high precision and a stable month cycle, which is of positive significance for monitoring the physiological state of trees. Samples were collected for 60 s every 1 h and 24 h a day on the same tree for a month. A total of 43,200 AE waveforms and 4.32 × 10^8^ sampling points were collected. We have carried out a measurement campaign on 4 typical tree species at Nanjing Forestry University (Robinia Pseudoacacia, Photinia serrulata, Pinus massoniana and Toona sinensis). The installation method of the WASN was as mentioned above: within a three-point line installation on the bark surface and Vaseline coupling. Finally, KT-80, a dual-function wood-moisture-measuring instrument from KLORTNER Technology, Italy, was used to calibrate the true value and predict and analyze the change in moisture content within a month. The four algorithms all use the AE waveform of four trees collected in the field, and the corresponding sample data set decomposed by ACMD as the training input. The MC predicted by different models and the calibrated real MC values of the 4 tree species are shown in Figure 12. The evaluation indexes of field results are depicted in Table 2.

## 4. Discussion

Accurate MC prediction is an important guarantee for monitoring the physiological activities of trees [28]. Changes in wind speed, photosynthetically active radiation, soil temperature and humidity have certain effects on tree trunk moisture, so it is considered important to add these factors before establishing the model in future work [29]. Compared with the traditional methods, which are complicated to install and cause irreversible damage to living trees, the multi-strategy joint optimization of IGWO-XGBoost proposed in this paper based on ACMD waveform decomposition draws the following conclusions:

1. Compared with the traditional VMD method, ACMD has a better decomposition effect for time-varying multimode AE signals, which can decompose the original AE signals step-by-step into stable and more representational subsequence groups. With the number of subsequence groups effectively reduced, the working time of the algorithm is shortened, and the prediction accuracy is improved.
2. By using Henon chaotic mapping, reverse learning strategy and firefly disturbance strategy, GWO was jointly optimized with multiple strategies, which enhanced the global search capability of GWO and improved its convergence speed. Standard test functions were used to compare and analyze the influence degree of these three improved strategies on IGWO.
3. By using multi-strategy joint optimization, IGWO optimizes the hyperparameters of XGBoost, and shorter iteration times and higher prediction accuracy are obtained compared with PSO, ACO and SOA.
4. Compared with the results of commercial MC measuring instruments [30], the proposed diagnostic method can stably and effectively characterize the actual MC change of tree trunks and has high applicability and accuracy in complex field conditions.

## Figures and Tables

**Table 1:** Improvement strategies of GWO.

Algorithm	Hénon Mapping	Reverse Learning Strategy	Firefly Perturbation
IGWO_1_	√	√	×
IGWO_2_	√	×	√
IGWO_3_	×	√	√
IGWO	√	√	√

**Table 2:** Results of MC recognition of four algorithms.

Algorithm	RMSE (%)	Roc-Auc	Iteration	Accuracy (%)
PSO-XGBoost	14.8	0.853 ± 0.025	24.36	90.27
ACO-XGBoost	12.2	0.897 ± 0.014	18.77	92.76
SOA-XGBoost	8.6	0.902 ± 0.012	17.5	93.12
IGWO-XGBoost	4.1	0.956 ± 0.008	8.32	96.75

## Data Availability

Not applicable.
